# The Predictive Value of a Modified Frailty Index on Perioperative Morbidity and Mortality Following Otologic Surgery

**DOI:** 10.1097/ONO.0000000000000029

**Published:** 2023-03-09

**Authors:** Kevin J. Quinn, Yuchi Ma, Matthew Carli, Daniel H. Coelho

**Affiliations:** 1 Department of Otolaryngology – Head and Neck Surgery, Virginia Commonwealth University School of Medicine, Richmond, Virginia; 2 Department of Biostatistics, Virginia Commonwealth University School of Medicine, Richmond, Virginia.

**Keywords:** Frailty, NSQIP, Otologic surgery

## Abstract

**Objectives:**

Recently, determinants of frailty have become an increasingly recognized perioperative risk stratification tool. This study examines the predictive value of a 5-factor modified frailty index (mFI-5) on perioperative morbidity and mortality in patients undergoing otologic surgery, with a subgroup analysis based on surgery site.

**Study Design:**

Cross-sectional analysis.

**Setting:**

National surgical quality improvement program dataset 2005–2019.

**Patients:**

Current procedural terminology (CPT) codes were used to identify patients undergoing all otologic surgeries.

**Interventions:**

Otologic surgeries as indicated by CPT codes, including external ear, middle ear/mastoid, implants, and inner ear/facial nerve subgroups.

**Main Outcome Measures:**

Primary outcomes examined in this study included rates of overall complications and life-threatening complications within 30 days after surgery. Overall complications included superficial surgical site infections (SSI), deep incisional SSI, readmission, deep vein thrombosis, life-threatening complications, and mortality. Life-threatening complications included those classified as Clavien-Dindo grade IV: cerebrovascular accident, mechanical ventilation for more than 48 hours, reintubation, pulmonary embolism, acute renal failure, cardiac arrest, and myocardial infarction.

**Results:**

A total of 16,859 patients who underwent otologic surgery were identified, resulting in a cohort that was 47.5% male with an average age of 47.6 years (17.1 SD). Multivariable regression analysis of the entire cohort demonstrated a score of 3 or more on the mFI-5 was independently predictive of all postoperative complications (odds ratio (OR): 2.02, *P* < 0.0001). However, subgroup analysis showed that only “external ear” surgery correlated with mFi-5 (OR 8.03, *P* = 0.013).

**Conclusions:**

Higher frailty scores as measured by the mFI-5 correlate with postoperative morbidity and mortality after otologic surgery, though subgroup analysis reveals an association only with cases performed on the external ear. These findings suggest that for most otologic surgery, the mFI-5 frailty score is not predictive of postoperative complications.

Over the past several decades, worldwide increases in life expectancy have resulted in a significant shift in the demographics of the human population—current estimates suggest that the average age of the global population will increase from 32.6 to 46.2 years between 2017 and 2100 (1). As the average age of the population increases, so does the incidence of comorbidities associated with increased age, including cancer, cardiovascular and respiratory disease, hypertension, arthritis, and diabetes (2). While all these factors can increase the risk of peri- and postoperative complications, recent efforts have looked beyond them to better define the factors that impact prognosis and outcomes in surgical patients. One of these factors that has gained substantial interest in recent years is frailty.

The concept of frailty describes a state of diminished physical, physiologic, and cognitive reserve and a loss of resistance to stressors caused by age-related deficits. It has been shown to be strongly associated with adverse outcomes in patients, even irrespective of age (3–5). In a wide variety of surgical subspecialties, frailty has been correlated with increased postoperative mortality in both low- and high-risk procedures (6).

The 5-factor modified-frailty index (mFI-5) is an easily calculated scoring system that has been proven to reflect frailty based on 5 comorbidities. These include diabetes mellitus, congestive heart failure, hypertension requiring medication, chronic obstructive pulmonary disease or current pneumonia, and nonindependent functional status. The utility of mFI-5 has been validated using the American College of Surgeons (ACS) National surgical quality improvement program (NSQIP) database in various surgical specialties (7–9). Within the field of otolaryngology, this has been studied in the context of anterior cranial fossa and skull base procedures, with a strong correlation found between increasing frailty scores and peri- and postoperative complications (7). However, the relationship between frailty and the occurrence of these complications in nonskull base otologic surgery, a substantially more common type of surgery than skull-base procedures, has yet to be examined. In this study, we aim to examine the NSQIP database to better understand the predictive role of mFI-5 in peri- and postoperative complications in otologic surgeries.

## MATERIALS AND METHODS

### Data Source

The ACS NSQIP database was queried for all patients undergoing otologic surgery between 2005 and 2019. This represents a deidentified compilation of postoperative 30-day morbidity and mortality outcomes from over 700 participating hospitals in the United States (10). In addition to demographic data, this database also provides pre- and perioperative risk factors that may be analyzed in correlation with postoperative outcomes.

The following current procedural terminology (CPT) codes were used to identify patients undergoing otologic surgeries, with subdivision into groups for external ear (69000–69399), middle ear/mastoid (69400–69700), implants (69710–69718, 69930), and inner ear/facial nerve (69720–69929, 69931–69979). These CPT codes were drawn from the NSQIP categories of “principal operating procedure,” “concurrent procedure,” and “other procedure,” to capture the broadest array of patients. This data was then analyzed in total as well as in the subgroups described above. For cases where multiple codes were present, only the most “invasive” code was included and generally prioritized as follows: inner ear/facial nerve > implants > middle ear/mastoid > external ear. As such each patient case was only counted once, such that a patient who for example underwent both a total intratemporal facial nerve decompression and a percutaneous osseointegrated auditory implant would be counted in the former category.

### Study Population and Variables

Demographic data collected include age, sex, body mass index (BMI), operative time, American Society of Anesthesiologists (ASA) class, and surgical wound class. Frailty scores were categorized using the 5-factor modified frailty index (mFI-5) specifically validated for the ACS NSQIP database and are calculated using the following variables: diabetes mellitus, congestive heart failure, hypertension requiring medication, chronic obstructive pulmonary disease or current pneumonia, and nonindependent functional status as defined by the NSQIP database (7). Patients with mFI-5 scores of three or more were grouped together due to small sample sizes.

### Primary Outcome Measures

Primary outcomes examined in this study included rates of overall complications and life-threatening complications within 30 days after surgery. Overall complications included superficial surgical site infections (SSI), deep incisional SSI, readmission, deep vein thrombosis, life-threatening complications, and mortality. Life-threatening complications included those classified as Clavien-Dindo grade IV: cerebrovascular accident, mechanical ventilation for more than 48 hours, reintubation, pulmonary embolism, acute renal failure, cardiac arrest, and myocardial infarction (11).

### Statistical Analysis

Descriptive statistics of baseline demographic variables were calculated, and subgroup comparisons were performed using student’s *t* test for continuous variables and chi-squared test for categorical variables. Firth’s logistic regressions were performed using R version 4.2.0. to examine associations between mFI-5 and outcome variables while controlling for age, gender, operation time, BMI, ASA class, and wound class. A *P* value less than 0.05 was regarded as statistically significant.

## RESULTS

A total of 16,859 patients who underwent otologic surgeries between 2005 and 2019 were included in the current study. Demographic data are available in Table 1. Approximately 69.8% of patients did not have any frailty-qualifying factor on the mFI-5 index, 22.0% had a scoring of 1, 7.4% had a scoring of 2, and 0.7% had a scoring of 3 or more. Most data for otologic procedures (94.6%) fell in the middle ear/mastoid category, with 2.9% in external ear, 1.8% in inner ear/facial nerve, and 0.7% in implants (Table 1).

**TABLE 1.** Clinical characteristics (n = 16,859)

	Overall (n = 16,861)		External ear (n = 481)		Middle ear/mastoid (n = 15,948)		Implants (n = 126)		Inner ear/facial nerve (n = 304)		
Demographics	Number	%	Number	%	Number	%	Number	%	Number	%	*P* value
Age											^a^
18–39	6060	35.9	81	16.8	5885	36.9	25	19.8	68	22.4	
40–64	7631	45.3	128	26.6	7290	45.7	53	42.1	159	52.3	
65–74	2211	13.1	123	25.6	2011	12.6	28	22.2	49	16.1	
≥75	959	5.7	149	31	762	4.8	20	15.9	28	9.2	
Sex											^b^
Male	8013	48.5	336	69.9	7474	46.9	50	60.3	152	50	
Female	8848	52.5	145	30.1	8474	53.1	76	39.7	152	50	
BMI, kg/m^2^											^c^
<18.5	535	3.2	10	2.1	519	3.3	1	0.8	5	1.6	
18.5–29.9	10567	62.7	313	65.1	9974	62.5	79	62.7	200	65.8	
30–34.9	3210	19	84	17.5	3041	19.1	24	19	61	20.1	
35–39.9	1454	5.6	46	9.6	1370	8.6	15	11.9	23	7.6	
≥40	1095	6.5	28	5.8	1044	6.5	7	5.6	15	4.9	
mFI-5 score											^d^
0	11771	69.8	213	44.3	11296	70.8	71	56.3	190	62.5	
1	3717	22	182	37.8	3421	21.5	36	28.6	77	25.3	
2	1253	7.4	78	16.2	1127	7.1	15	11.9	33	10.9	
3+	120	0.7	8	1.7	104	0.6	4	3.2	4	1.3	

^a^Significant differences in age found between external ear vs middle ear/mastoid (*P* < 0.001) and external ear vs inner ear/facial nerve (*P* < 0.001) subgroups.

^b^Significant differences in gender found between external ear vs middle ear/mastoid (*P* < 0.001), external ear vs implants (*P* < 0.001), and external ear vs inner ear/facial nerve (*P* < 0.001) subgroups.

^c^Significant differences in BMI found between external ear vs middle ear/mastoid (*P* = 0.045).

^d^Significant differences in mFI-5 score found between external ear vs middle ear/mastoid (*P* < 0.001), middle ear/mastoid vs implants (*P* < 0.001), and middle ear/mastoid vs inner ear/facial nerve (*P* < 0.001).

ASA indicates American Society of Anesthesiologists; BMI, body mass index; mFI-5, 5-factor modified frailty index.

**FIG. 1.**
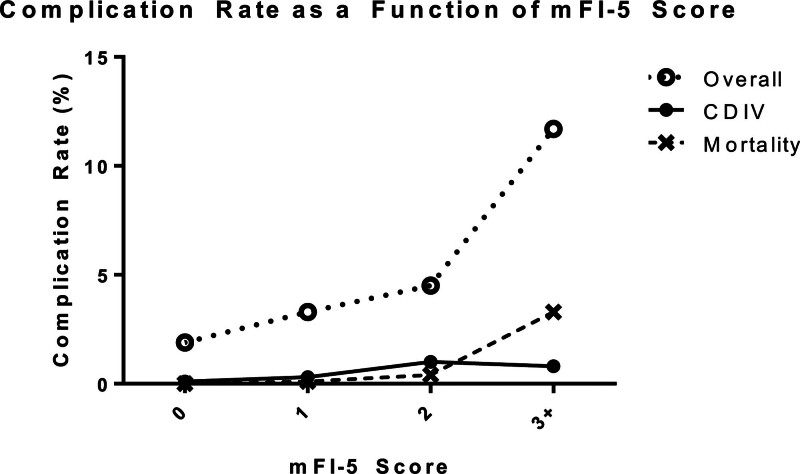
Rates of complications following otologic surgery as a function of mFI-5 score. Overall complications: SSI, deep SSI, DVT, CDIV complications, death; CDIV complications: Stroke, postoperative ventilator requirement >48 h, reintubation, acute renal failure, pulmonary embolism, cardiac arrest, myocardial infarction. Overall complications increased significantly between mFI-5 scores 0 vs 1 (*P* = 0.0001) and 2 vs 3+ (*P* = 0.0016). CDIV complications increased significantly between mFI-5 scores 0 vs 1 (*P* = 0.0159) and 1 vs 2 (*P* = 0.0071). Mortality increased significantly between mFI-5 scores 1 vs 2 (*P* = 0.0172) and 2 vs 3+ (*P* = 0.0013). CDIV indicates Clavien-Dindo grade IV; DVT, deep venous thrombosis; mFI-5, 5-factor modified frailty index; SSI, superficial surgical site infection.

Overall complication rate of all otologic procedures was 2.5%, with the most common complication being readmission (1.3%). The rate of life-threatening complications was 0.3%. Details of the complications rates associated with otologic surgeries is described in Table 2. Increases in rates of overall complications and 30-day mortality were observed with the increase in mFI-5 score (Fig. 1). On Firth’s logistic regression, a mFI-5 score of 3 or more was predictive of overall postoperative complications (odds ratio (OR) = 2.15, 95% CI: 1.12–3.89, *P* = 0.0227) as well as 30-day mortality (OR = 9.68, 95% CI: 1.77–57.06, *P* = 0.0093) (Table 3). Life-threatening complications were not correlated with mFI-5 scores.

**TABLE 2.** Rates of complications after otologic surgeries (n = 16,859)

	Overall (n = 16,861)	External ear (n = 481)	Middle ear/mastoid (n = 15,948)	Implants (n = 126)	Inner ear/facial nerve (n = 304)
	%	%	%	%	%
Overall complications	2.5	9.4	2.1	11.1	7.6
Readmission	1.3	4.4	1.1	4.0	5.6
Superficial SSI	0.9	2.3	0.8	5.6	0.7
Life-threatening complications	0.3	2.3	0.2	1.6	1.6
Deep incisional SSI	0.2	1.2	0.2	1.6	1.0
Deep vein thrombosis	0.1	0.2	0.0	0.8	0.7
Cerebrovascular accident	0.1	0.4	0.0	0.0	0.3
Mechanical ventilation >48 h	0.1	0.6	0.1	0.0	0.3
Reintubation	0.1	1.2	0.1	0.0	0.0
Myocardial infarction	0.1	0.6	0.0	0.8	0.0
Mortality	0.1	0.8	0.1	0.0	0.3
Pulmonary embolism	0.0	0.0	0.0	0.8	1.0
Acute renal failure	0.0	0.2	0.0	0.0	0.0
Cardiac arrest	0.0	0.0	0.0	0.0	0.0

SSI indicates surgical site infection.

**TABLE 3.** Firth’s regressions of mFI-5 and rates of complications with subgroup analysis

	Overall complications		CDIV		Mortality	
	OR (95% CI)	*P*	Number	*P*	Number	*P*
Surgical site						
All sites (N = 16,859)						
mFI-5 = 1	1.00 (0.77–1.29)	0.9934	0.71 (0.31–1.61)	0.4154	0.37 (0.06–2.11)	0.2586
mFI-5 = 2	1.04 (0.73–1.47)	0.8111	1.37 (0.58–3.27)	0.4689	1.63 (0.37–8.27)	0.5201
mFI-5 = 3+	**2.15 (1.12–3.89**)	**0.0227**	0.84 (0.08–4.10)	0.8501	**9.68 (1.77–57.06**)	**0.0093**
External ear (N = 481)						
mFI-5 = 1	0.46 (0.19–1.09)	0.0773	0.50 (0.09–2.73)	0.4146	0.13 (0.00–1.95)	0.1434
mFI-5 = 2	0.67 (0.24–1.81)	0.4311	1.81 (0.29–11.46)	0.5218	0.12 (0.00–2.82)	0.1978
mFI-5 = 3+	**8.03 (1.56–45.42**)	**0.0130**	1.55 (0.01–28.61)	0.8103	8.05 (0.83–132.02)	0.0872
Middle ear/mastoid (N = 15,948)						
mFI-5 = 1	1.09 (0.82–1.44)	0.5704	1.15 (0.38–3.57)	0.8010	0.76 (0.09–8.98)	0.8059
mFI-5 = 2	1.02 (0.68–1.52)	0.9042	2.62 (0.85–8.58)	0.0945	3.30 (0.54–36.99	0.2062
mFI-5 = 3+	1.56 (0.64–3.28)	0.3040	2.57 (0.24–15.02)	0.3778	7.05 (0.47–105.90)	0.1435
Implants (N = 126)						
mFI-5 = 1	1.39 (0.30–6.60)	0.6704	3.00 (0.15–412.81)	0.4734	1.04 (0.00–755.82)	0.9842
mFI-5 = 2	1.54 (0.20–10.63)	0.6642	2.08 (0.00–1135.44)	0.7557	1.61 (0.01–4339.41)	0.8292
mFI-5 = 3+	2.24 (0.13–28.96)	0.5500	1.72 (0.00–418.58)	0.7928	5.06 (0.01–71375.71)	0.5590
Inner ear/facial nerve (N = 304)						
mFI-5 = 1	1.20 (0.38–3.82)	0.7525	0.33 (0.03–2.43)	0.2828	4.11 (0.00–10065.87)	0.6222
mFI-5 = 2	2.13 (0.55–8.14)	0.2713	0.09 (0.00–1.67)	0.1172	13.66 (0.00–21451026.65)	0.3837
mFI-5 = 3+	2.19 (0.15–22.44)	0.5359	0.08 (0.00–4.24)	0.2394	**250.12 (1.38–71296191792.78**)	**0.0348**

Bold values represent *p* <0.05.

CDIV indicates Clavien-Dindo grade IV complications; CI, confidence interval; mFI-5, 5-factor modified frailty index; OR, odds ratio.

On surgical site subgroup analysis, the odds of overall complications were significantly increased with a mFI-5 score of 3 or more in external ear surgeries (OR = 8.03, 95% CI: 1.56–45.42, *P* = 0.0130) and the odds of 30-day mortality were significantly increased with a mFI-5 score of 3 or more in inner ear and facial nerve surgeries (OR = 250.12, 95% CI: 1.38–71296191792.78, *P =* 0.0348) (Table 3). No other complications were correlated with mFI-5 scores in other surgical sites.

## DISCUSSION

The importance of risk stratification in medicine has been increasingly recognized over the past several decades. Efforts to appropriately stratify patients span all fields of healthcare, including both the medical fields and their surgical counterparts (12,13). This has been driven in large part by a prevailing healthcare environment that increasingly prioritizes efficient utilization of resources and encourages conservation of resources. Beyond this, risk stratification enhances physicians’ abilities to counsel patients during the informed consent process, which in turn offers the latter greater agency and autonomy in their healthcare decisions.

One of the primary modalities utilized in risk stratification is frailty. This term, defined as “the quality or state of being physically weak,” has been expanded within healthcare to “a state of diminished physiological reserve that limits resolution of homeostasis following a stressor” (14,15).

Efforts to quantify frailty have evolved over the years, with many researchers implementing an “accumulation of deficits” model to develop their scoring systems (16). One of the early implementations of this approach was the Canadian Study of Health and Aging, which developed a 70-item scale incorporating a variety of measures including grip strength, cognitive function, and comorbid conditions that was predictive of both morbidity and mortality (17). As many of these variables are not routinely collected in all clinical settings, an alternative scoring metric was developed using the NSQIP database from the ACS. Over time this has evolved from an initial 16 variables (corresponding with 11 factors from the CSHA frailty index) to the 5 variables that are used in the modified Frailty Index, or mFI-5 (8).

While the mFI-5 has not previously been studied in the context of the spectrum of otologic surgery, other disciplines within the field of otolaryngology have employed it as a means of risk stratification. Cuccolo et al (18) found that mFI-5 scores of 3 or more were predictive of all-cause complications following head and neck pedicled flap reconstruction. Panayi et al (19) performed a comprehensive review of the NSQIP database for microvascular reconstructive surgeries performed in the head and neck. They found that increasing frailty scores were significantly associated with increased mortality rates, longer hospitalization, and increased readmissions, including in subgroup analysis by age. Similar findings have been reported in the context of facial fracture repair (20).

In the field of neurotology/skull base surgery, Henry et al (7) examined the relationship between mFI-5 scores and postoperative outcomes for patients undergoing skull base surgery and noted both a significant and stepwise correlation between frailty scores and postoperative morbidity, mortality, and length of stay for middle, posterior, and multifossa surgeries. Likewise, a cohort of 218 patients undergoing vestibular schwannoma resection confirmed these findings, with frailty scores of 1 or more associated with increased hospital length of stay but not with the incidence of postoperative complications (21).

The present study similarly demonstrates a correlation between overall postoperative complications and mortality and mFI-5 scores of 3 or more when *all* surgical sites were included. However, subgroup analysis by surgical site revealed that only external ear surgeries maintained this significance. While this stands in contrast with the aforementioned studies, when the external ear cohort is eliminated, the data are in agreement with the work of Gordon et al (22), who studied the mFI-5 in a cohort of over 500 cochlear implant patients implanted at a single institution. They found no difference in complications between patients with an mFI-5 score of 0 and those with a score of 1 or more. Taken together, such would suggest that mFI-5 is not predictive of risk in otologic surgeries except in surgery primarily involving the external ear.

The reasons why mFI-5 correlates only with the external ear procedures and not other otologic procedures are not readily apparent from the data. Theoretically clinicians may (incorrectly) assume that “superficial” surgery of the auricle and ear canal is less invasive than “deeper” surgeries of the middle ear and mastoid, thereby offering surgery to patients who otherwise would have been deemed poor surgical candidates. Along those lines, surgeons may elect to use local/monitored anesthesia for external ear cases preferentially over middle ear/mastoid cases (or vice versa) thereby introducing bias. Unfortunately, there were no data availably detailing the type of anesthesia (general versus local) administered by case. An alternative explanation is the contribution of oncologic patients to the external ear subgroup. All patients undergoing lateral temporal bone resections or auriculectomies would be more likely to have an indication of carcinoma than other temporal bone subsites. A diagnosis of cancer is independently associated with an increased risk of surgical complications (23). In addition, superficial injuries to the pinna requiring repair may be secondary to more serious head trauma, itself associated with substantial morbidity and mortality. Unfortunately, the NSQIP database (and the imputed mFI-5) do not includes etiology (eg, oncologic, traumatic)—another potential shortcoming of both.

Nonetheless, nearly 95% of patients in this study did not have surgery of the external ear which is likely an accurate reflection of the prevalence of these types of surgery within otology. Thus, for the majority of patients undergoing otologic surgery, complications are rare and therefore the mFI-5 may not serve as accurate marker of risk stratification.

Interestingly, the facial nerve/inner ear cohort did have a higher associated 30-day mortality compared with other subsites. Although purely theoretical based on the limited clinical information in the database, cases of post-traumatic inner ear/facial nerve surgery may have sustained other non-temporal bone injuries that may have contributed to their mortality.

Fortunately, morbidity and mortality following otologic procedures are relatively rare. Thus, to achieve any statistically meaningful conclusions regarding risks, “big data” such as those found in the NSQIP become indispensable. Yet, such analyses are not without limitations. Those associated with this study are primarily due to its retrospective nature, as well as the cohort from which it is drawn. The NSQIP database only includes information collected within 30 days of surgery, limiting its utility in estimating the sequalae of surgical interventions. There is also a possibility of selection bias given that academic centers make up the majority of NSQIP-participating institutions. While our total cohort of patients was sizable, there was a relatively low incidence of mFI-5 scores that were 3 or more, as well as a low incidence of complications. These factors in conjunction resulted in large confidence intervals in several of our analyses and could limit our ability to detect a more subtle statistically significant difference. Finally, our prioritization algorithm of codes for cases in which multiple subsites were involved may have unintentionally resulted in an inaccurate representation of the subgroup cohorts. It was decided a priori to data collection to include only 1 CPT. Electing to include more than one would give an overestimation of the contribution of case duration, which is likely substantially lower in cases with multiple codes performed under the same anesthetic. Ultimately, the effect is likely minimal as most cases were affiliated with only one subsite. In addition, the inclusion of multiple CPTs is a more accurate reflection of real-life where a substantial number of patients undergo multiple simultaneous procedures.

## CONCLUSION

Higher frailty scores as measured by the mFI-5 correlate with post-operative morbidity and mortality after otologic surgery, though subgroup analysis reveals an association only with cases performed on the external ear. These findings suggest that for most otologic surgery, the mFI-5 frailty score is not predictive of postoperative complications. Surgeons should be aware that complications from surgeries of the external ear may correlate with frailty and counsel patients and families accordingly.

## FUNDING SOURCES

None declared.

## CONFLICT OF INTEREST STATEMENT

None declared.

## DATA AVAILABILITY STATEMENT

The datasets generated during and/or analyzed during the current study are publicly available.
